# Donor Lymphocyte Infusion and Molecular Monitoring for Relapsed Myelofibrosis After Hematopoietic Cell Transplantation

**DOI:** 10.1097/HS9.0000000000000921

**Published:** 2023-06-30

**Authors:** Nico Gagelmann, Christine Wolschke, Anita Badbaran, Dietlinde Janson, Carolina Berger, Evgeny Klyuchnikov, Francis Ayuk, Boris Fehse, Nicolaus Kröger

**Affiliations:** 1 Department of Stem Cell Transplantation, University Medical Center Hamburg-Eppendorf, Hamburg, Germany

## Abstract

Hematopoietic cell transplantation (HCT) is a curative approach for myelofibrosis patients, but relapse is a major cause of treatment failure. We investigated the effect of donor lymphocyte infusion (DLI) in 37 patients with molecular (n = 17) or hematological relapse (n = 20) after HCT. Patients received median of 2 (range, 1–5) cumulative DLI (total of 91 infusions). Median starting dose was 1 × 10^6^ cells/kg, escalated by half-log ≥6 weeks if no response nor graft-versus-host disease (GvHD) occurred. Median time to first DLI was 40 weeks for molecular relapse versus 145 weeks for hematological relapse. Overall molecular complete response (mCR) at any time was 73% (n = 27) and was significantly higher for initial molecular relapse (88%) versus hematological relapse (60%; *P* = 0.05). The 6-year overall survival was 77% versus 32% (*P* = 0.03). Acute GvHD 2–4 occurred in 22% and half of the patients achieved mCR without any GvHD. All patients who relapsed from mCR achieved after first DLI could be salvaged with subsequent DLI, showing long-term survival. No second HCT was needed for molecular relapse versus 6 for hematological relapse. This comprehensive and largest study to date suggests molecular monitoring together with DLI as standard of care and a crucial approach to achieve excellent outcomes in relapsed myelofibrosis.

## Graphical abstract

**Figure:**
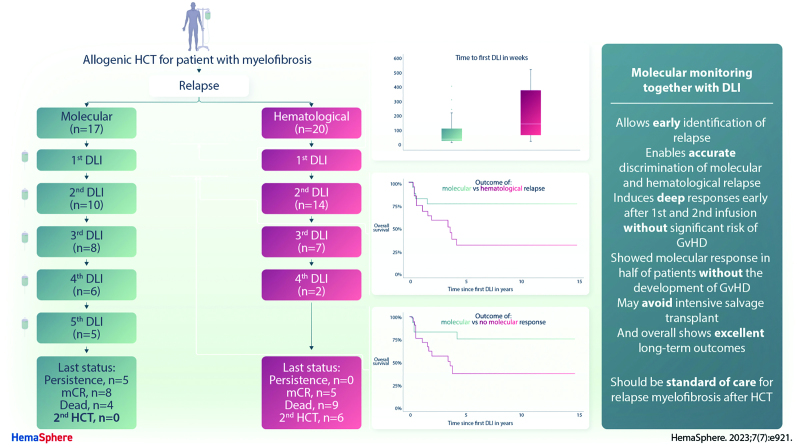

## INTRODUCTION

Myelofibrosis is a clonal myeloproliferative neoplasm with a heterogenous clinical presentation and outcome,^1^ with survival lasting from months to decades.^2^ Despite the recent advances and the promising introduction of new targeted therapies,^3^ allogeneic hematopoietic cell transplantation (HCT) remains the only curative treatment option, which is, however, associated with relevant morbidity and mortality.^4,5^

About 10%–30% of myelofibrosis patients who undergo allogeneic HCT will eventually experience relapse within 5 years.^6,7^ The median time to relapse was previously shown to be ~7 months, with a median overall survival (OS) from the time of relapse of ~2 years, being significantly worse for those who relapse early.^7,8^ While significant improvement has been achieved in stratifying patients according to their risk for posttransplant mortality,^9–11^ preventing and treating relapse remains an unmet clinical need.

The concept of transferring donor T cells aims at reinforcing a graft-versus-leukemia effect targeting the recurring malignant cells, which has mainly been shown for acute and chronic leukemias.^12^ Previous small sample studies also indicated a feasible and strong graft-versus-myelofibrosis effect of donor lymphocyte infusion (DLI) for elapsed patients.^13–15^ Additionally, in more recent years, molecular monitoring posttransplant was able to detect minimal measurable disease, which predicts a high risk of subsequent hematological relapse after allogeneic HCT and can be used for early intervention with DLIs.^16,17^

Here, we evaluated the so far largest cohort to date of myelofibrosis patients with relapsed disease after HCT who received DLI as standard of care posttransplant management for either molecular relapse or hematological relapse. The aim of this study was to compare efficacy regarding achievement of molecular complete response (mCR), incidence of acute and chronic graft-versus-host disease (GvHD), and survival between both approaches.

## METHODS

### Transplant platform and DLI algorithm

We included patients with primary or secondary myelofibrosis (evolving from either polycythemia vera or essential thrombocythemia) who underwent first or second HCT and received DLI for molecular or hematological relapse between 2002 and 2019 in the Department of Stem Cell Transplantation at the University Medical Center Hamburg/Germany. Hematological relapse occurred more frequently in earlier years before the standardized implementation of molecular monitoring according to the institutional guidelines. All patients received reduced intensity busulfan-fludarabine conditioning, with anti T-lymphocyte globulin (ATLG) as GvHD prophylaxis as recently reported.^18^ Patients who received a second transplantation were censored at day of second transplantation. Patients with transformed acute leukemia at the time of transplant or relapse were excluded from the study.

**Figure 1.**
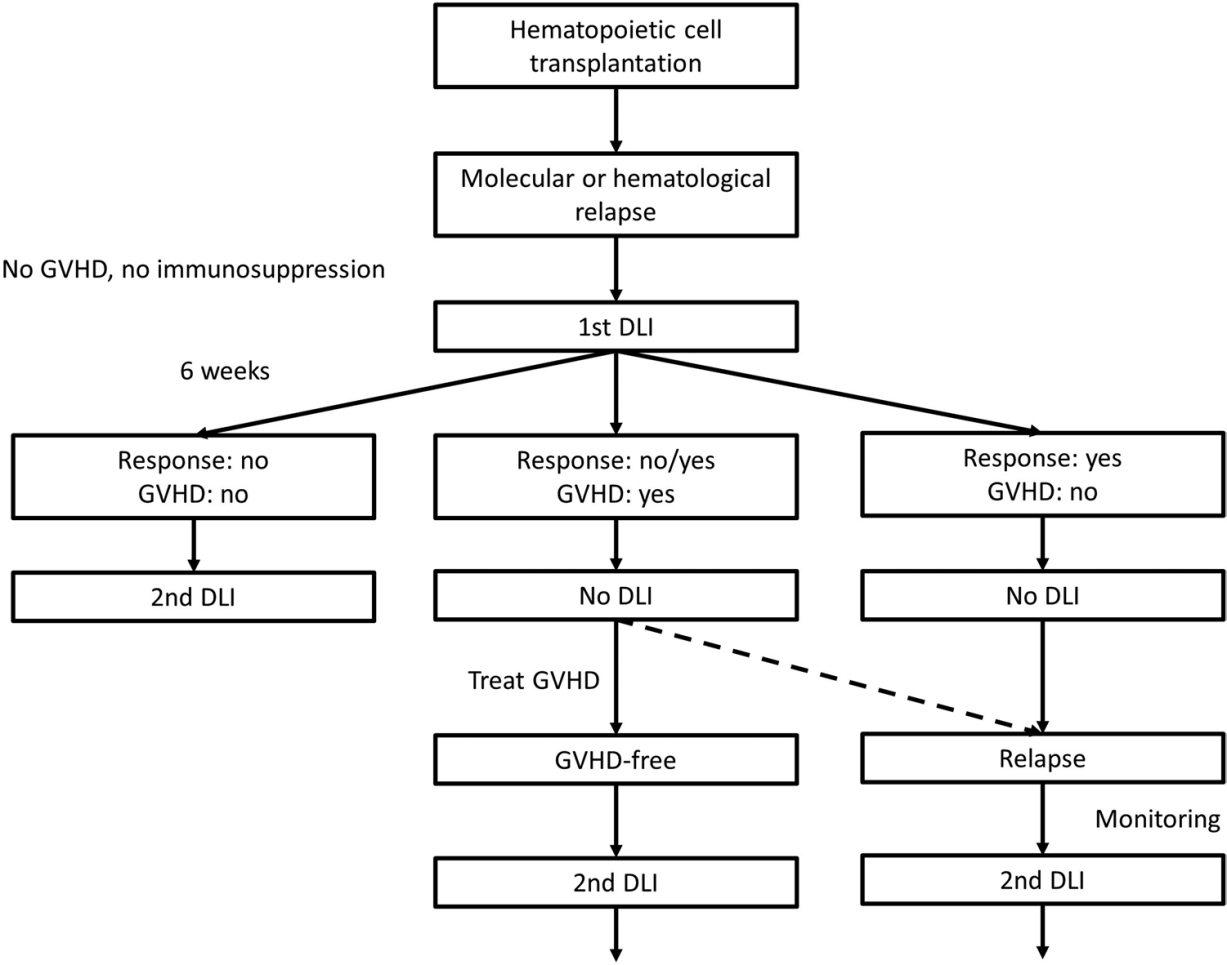
**Algorithm for monitoring and treatment of relapsed myelofibrosis after hematopoietic cell transplantation.** DLI = donor lymphocyte infusion; GVHD = graft-versus-host disease.

Patients were eligible to receive DLI if they were free of GvHD and immunosuppressive therapy (Figure 1). In relapsed patients who were still receiving prophylactic immunosuppression, immunosuppressive medication was stopped at least 4 weeks before the administration of dose-escalated DLI. If no GvHD or response occurred within 6 weeks, another escalating dose (half-log) of DLI was administered. The usual starting dose after HLA identical sibling HCT was 1 × 10^6^ CD3+ cells per kg body weight (kgBW) and 5 × 10^5^ CD3+cells after unrelated donor HCT resulting in a median dose of the first DLI of 1 × 10^6^/kgBW (range, 1 × 10^5^–1 × 10^7^) followed by a subsequent half-log escalated dose after at least 6 weeks if there was no response and no GvHD (Figure 1). CD4-selected DLI were used in patients with high risk for or present infection at the time of relapse.

### Monitoring

Follow-up of patients after allograft included molecular monitoring (including chimerism analysis) every month (until day 360 posttransplant) and with half-year routine visits in the following 2 years and yearly visits after 3 years thereafter, while bone marrow histology was performed after engraftment, on day 100 and 360 posttransplant, and thereafter only if there were signs of relapse. We applied *JAK2V617F*-, *CALR*-, and *MPL*-specific quantitative or digital polymerase chain reactions (PCRs) as described recently (sensitivity for both: 0.01%).^16,19^ At the time of transplantation, next-generation sequencing was done using previously published methods,^20^ and the following myelofibrosis-associated genes were sequenced: *JAK2, CALR, MPL, ASXL1, IDH1/2, CBL, DNMT3A, TET2, SF3B1, SRSF2, U2AF1, EZH2, TP53, NRAS, KRAS, RUNX1,* and *FLT3.*

### Definition of relapse and GvHD

Hematological relapse of myelofibrosis and response to therapy were defined according to the existing criteria,^4,6,21^ such as progressive splenomegaly, loss of complete response, morphological relapse (increasing bone marrow fibrosis, increase in age-adjusted cellularity and abnormal M:E ratio, megakaryocytic abnormalities typical of myelofibrosis such as pleomorphism, hyperchromasia, cloud-like nuclei, and megakaryocytic clusters), decrease in conventional donor chimerism, and worsening anemia. Additionally, detection of driver mutation in peripheral blood in previously negative patients was defined as molecular relapse. Molecular CR was defined as 2 subsequent negative driver mutation blood samples within at least 4-week interval in previously positive patients, or consecutive full highly sensitive chimerism in triple-negative patients. Acute GvHD was graded I–IV according to the standard criteria.^22^ Chronic GvHD was evaluated as mild or moderate disease according to the National Institutes of Health criteria.^23^

### Statistical analysis

Main end points were mCR, the occurrence of acute and chronic GvHD, event-free survival (EFS), and OS. Patient characteristics were described with median and range for continuous variables and frequencies for categorical variables. EFS was defined as the time between start of DLI treatment until relapse, progression, death from any cause or another subsequent allogeneic stem cell transplantation. Continuous variables were investigated with Mann-Whitney *U* test, Kruskal-Wallis test, and categorical variables with χ^2^ test. Survival curves were calculated with the Kaplan-Meier method and tested by log-rank-test; a *P* value of 0.05 or less was rated as significant. Median follow-up was calculated using the reverse Kaplan-Meier method. To assess the cause-specific effect of factors on each end point, we used the Cox proportional hazards model to estimate hazard ratios (HRs), evaluating landmark approaches from first DLI as well as continuous and time-dependent analysis. The proportional hazards assumption was verified using graphical methods.

All tests were 2-sided, with the type I error rate fixed at α = 0.05. Only results of the final models are presented with respect to a reference category (HR, 1.00) together with the 95% confidence interval (CI) and *P* values. All analyses were performed using R version 4.0.5 (R Foundation for Statistical Computing, Vienna, Austria) using the following packages: ggalluvial, ggplot2, tidyverse, survival, cmprsk, prodlim, and rms.

## RESULTS

### Patients

**Figure 2.**
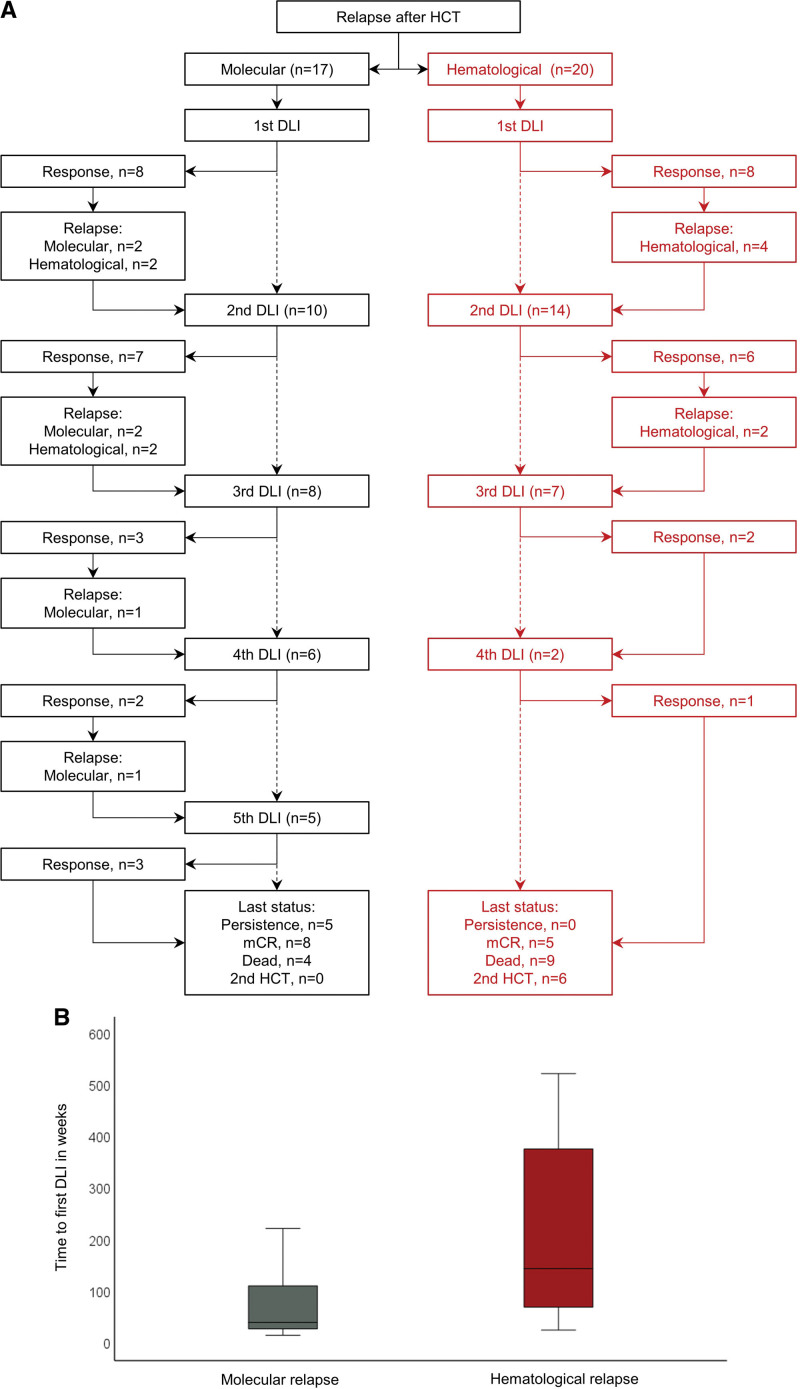
**Donor Lymphocyte Infusion for molecular and hematological relapse after allogeneic stem cell transplantation for myelofibrosis.** (A) Flow diagram for molecular or hematological relapse with numbers of DLI, response and subsequent relapse. (B) Time to first DLI according type of relapse. DLI = donor lymphocyte infusion; HCT = hematopoietic stem cell transplantation.

We included 37 relapsed patients of whom 22 patients had primary myelofibrosis (60%) and 30 patients had *JAK2* driver mutation genotype (81%). The median time from HCT to relapse was 19 months (range, 2–118 months). Seventeen patients received first DLI for molecular relapse and 20 patients received first DLI for hematological relapse after HCT (Table 1 and Figure 2A).

**Table 1:** Patient Characteristics

		Relapse	
Characteristic	Total (n = 37)	Molecular (n = 17)	Hematological (n = 20)
Age at transplantation, y	56 (32–74)	56 (33–74)	56 (32–72)
Transplantation			
First	33 (89)	14 (82)	19 (95)
Second	4 (11)	3 (18)	1 (5)
Diagnosis			
Primary myelofibrosis	22 (60)	10 (59)	12 (60)
Post-ET	7 (19)	3 (18)	4 (20)
Post-PV	8 (21)	4 (23)	4 (20)
DIPSS			
Intermediate-1	10 (27)	7 (42)	3 (15)
Intermediate-2	17 (46)	5 (29)	12 (60)
High	10 (27)	5 (29)	5 (25)
Karnofsky performance status			
90–100	23 (62)	9 (53)	14 (70)
70–80	14 (38)	8 (47)	6 (30)
Driver mutation status			
*CALR*	4 (11)	3 (18)	1 (5)
*JAK2*	30 (81)	14 (82)	16 (80)
Triple negative	3 (8)	0	3 (15)
High molecular risk^*a*^	10(44)	3 (27)	7 (58)
Donor type			
Related	11 (30)	6 (35)	5 (25)
Unrelated	26 (70)	11 (65)	15 (75)
Cumulative number of DLI/patient, median (range)	2 (1–5)	2 (1–5)	2 (1–4)
Total number of infusions	91	50	41
Dose of CD3 + cells/kgBW, median (range)			
First DLI	1 × 10^6^ (5 × 10^5^–1 × 10^7^/l)	1 × 10^6^ (5 × 10^5^–5 × 10^6^/l)	1 × 10^6^ (5 × 10^5^–1 × 10^7^/l)
Second DLI	5 × 10^6^ (1 × 10^6^–1 × 10^8^/l)	5 × 10^6^ (1 × 10^6^–1 × 10^7^/l)	5 × 10^6^ (1 × 10^6^–6 × 10^8^/l)
Time to 1st DLI in weeks	68 (15–523)	40 (15–407)	145 (25–523)

^*a*^Twenty-three evaluable patients (12 with hematological relapse and 11 with molecular relapse).

BW = body weight; DIPSS = dynamic International Prognostic Scoring System; DLI = donor lymphocyte infusions; ET = essential thrombocythemia; PV = polycythemia vera.

Thirty-three patients (89%) received DLI after first transplantation while the remaining 4 received DLI after second transplantation. Three patients received CD4-selected DLI. A total of 91 escalating infusions were applied, and the number of cumulative infusions ranged from 1 to 5 per patient, and of all 37 patients, the majority (57%) received only 1 (n = 11) or 2 DLI (n = 10), while 19% (n = 7) received 3 DLI, 11% (n = 4) received 4 DLI, and 13% (n = 5) received 5 cumulative DLI. Median time from transplantation to first DLI was 68 weeks (range, 15–523 weeks), from first to second DLI 6 weeks (range, 6–323 weeks), from second to third DLI 6 weeks (range, 6–220 weeks), from third to fourth DLI 6 weeks (range, 6–103 weeks), and from fourth to fifth DLI 6 weeks (range, 6–276 weeks). Longer duration to next DLI resulted from patients who first achieved mCR but eventually relapsed and received subsequent DLI.

There was no significant difference in number of DLI when comparing different indication groups for first DLI. However, all patients who received 5 DLIs initially belonged to the molecular relapse group.

### Overall response

Overall mCR rate (number of patients who achieved mCR at any time) was 73% (n = 27), and 10 patients (27%) never achieved mCR (Table 2). After first DLI, 16 of all 37 patients (43%) achieved an mCR. No significant association of DLI dose and achievement of mCR after first DLI was observed (*P* = 0.47). Patients who achieved mCR after first DLI showed less likelihood of relapse (*P* = 0.001) and thus necessity for subsequent DLIs (*P* < 0.001). There was no difference in overall response to DLI between patients after first versus second HCT (73% versus 75%). Time to relapse appeared to be associated with overall response (*P* = 0.08), showing higher likelihood of mCR for earlier relapse.

**Table 2:** Results

		Relapse		
Outcome	Total (n = 37)	Molecular (n = 17)	Hematological (n = 20)	*P*Value
Overall mCR, n (%)	27 (73)	15 (88)	12 (60)	0.05
Cumulative number of DLIs to achieve mCR, median (range)	2 (1–5)	2 (1–5)	3 (1–4)	0.41
Cumulative median T-cell dose to achieve mCR ×10^6^ (CD3+/kg)	3.5 (0.5–101)	3 (0.5–61)	5 (0.5–101)	0.53
Patients with 2nd HCT, n (%)	6 (16)	0 (0)	6 (30)	0.004
Overall acute GvHD II–IV, n (%)	8 (22)	3 (18)	5 (25)	0.59
Overall acute GvHD grade III/IV, n (%)	4 (11)	2 (12)	2 (10)	0.49
Overall chronic GvHD, n (%)	8 (22)	6 (35)	2 (10)	0.06
mCR without GvHD, n (%)	14 (38)	7 (52)	7 (58	0.70
EFS at 5 y, % (95 CI)	40 (23–57)	59 (36–82)	25 (4–46)	0.11
OS at 5 y, % (95 CI)	53 (36–70)	77 (57–97)	32 (10–54)	0.03

EFS = event-free survival; GvHD = graft-versus-host disease; mCR = molecular complete response; OS = overall survival; TRM = treatment-related mortality.

Donor chimerism was significantly associated with mCR after first DLI, showing median donor chimerism of 99.7% (range, 13%–100%) in patients with mCR versus 78.7% (range, 0%–100%) for patients without mCR (*P* = 0.003). The effect was mediated by type of relapse (*P* = 0.05), showing median donor chimerism after first DLI of 96.5% (range, 4%–100%) for molecular relapse versus 85% (range, 0%–100%) for hematological relapse. The prognostic utility, using concordance index, was 0.63 for molecular response and 0.51 for donor chimerism. The same was seen for second DLI, showing median donor chimerism of 99.9% (range, 24%–100%) in patients with mCR versus 36.6% (range, 0%–100%) for patients without mCR (*P* = 0.004).

### Response for molecular versus hematological relapse

Next, we compared outcomes of patients according to the type of initial relapse after HCT (molecular versus hematological). Median time from HCT to first DLI was shorter for patients with molecular relapse (40 weeks) compared with hematological relapse (145 weeks; Figure 2B). Patients who showed initial molecular relapse after HCT appeared to be more likely to achieve mCR at any time (88%; 15/17) versus hematological relapse (60%; *P* = 0.05). Median number of cumulative DLI for responders was 2 (range, 1–5) for molecular relapse and 3 (range, 1–4) for hematological relapse. Cumulative dose of all responders was 15 × 10^6^ cells/kgBW (range, 5 × 10^5^–1 × 10^7^) for patients with initial molecular versus 15 × 10^6^ cells/kgBW (range, 5 × 10^5^–6 × 10^8^) for hematological relapse, respectively.

Forty-seven percent of patients (8/17) with molecular relapse showed mCR after first DLI compared with 40% of patients (8/20) with initial hematological relapse. Median dose of first DLI for responders was 1 × 10^6^/kgBW (range, 5 × 10^5^–3.7 × 10^6^) for molecular relapse versus 1 × 10^6^ (range, 5 × 10^5^–1 × 10^7^) for hematological relapse. Importantly, significantly more patients with initial molecular relapse after HCT who did not achieve mCR after first DLI eventually achieved mCR with subsequent DLI (78%; 7/9) compared with only 33% (4/12) of patients with hematological relapse after HCT and no response after first DLI (*P* = 0.04). After second DLI, mCR rate was 58% for patients with initial molecular relapse compared with 43% for patients with initial hematological relapse.

Of the 17 patients with molecular relapse, 13 (76%) also showed lack of full donor chimerism at the time of first DLI. mCR was associated with achievement of full donor chimerism (*P* < 0.001), and 5 patients had persistent disease (lack of full donor chimerism and no mCR) but were alive at last follow-up after a total of 1 infusion (n = 1), 2 infusions (n = 1), 4 infusions (n = 1), and 5 infusions (n = 2), respectively.

### Acute and chronic GvHD

In terms of acute GvHD, only 8 overall events occurred (Table 3) after a total number of 91 infusions (9%). Incidence of acute GvHD II–IV and grade III/IV with respect to all patients was 22% (8/37) and 11% (4/37). Acute GvHD was not associated with overall mCR. No association of acute GvHD and T-cell dose was observed (*P* = 0.98). There were 2 cases of severe acute GvHD after first DLI (grade III of the liver and IV of the skin, liver, and gastrointestinal, respectively). Both patients achieved mCR after first DLI (*P* = 0.09) and received a DLI dose of 5 × 10^6^/kgBW compared with a median of 1 × 10^6^/kgBW for patients without acute GvHD, while the patient with grade IV acute GvHD died of treatment-related mortality due to sepsis with multiorgan failure. All cases were steroid-responsive and resolved.

**Table 3:** Events and Severity of Graft-versus-host Disease After DLI

DLI	Acute GvHD	Chronic GvHD
1st	2 (grade III: liver; grade IV: skin, liver, GI)	1 (moderate: skin, eyes)
2nd	4 (2 grade II: skin; 2 grade III: skin)	3 (1 mild: skin; 2 moderate: skin, mouth eyes)
3rd	0	1 (mild: skin)
4th	1 (grade II: skin)	4 (mild: skin)
5th	1 (grade II: liver)	0
Total	8	8

DLI = donor lymphocyte infusion; GI = gastrointestinal; GvHD = graft-versus-host disease.

In terms of chronic GvHD, only 8 overall cases occurred (9%; Table 3) and appeared to be associated with response (*P* = 0.05). All patients with chronic GvHD achieved mCR, while 66% (19/29) of those without chronic GvHD achieved mCR. No association was observed for first or second DLI dose with occurrence of chronic GvHD. Other factors such as time between allograft and DLI, HLA mismatch donor transplantation, or CD4-selected products did not influence the incidence of acute or chronic GvHD. All 3 patients receiving CD4-selected products achieved mCR after first DLI without GvHD and were alive at last follow-up.

In terms of other toxicities, only 1 patient showed pancytopenia co-occurring to acute GVHD grade IV. However, bone marrow examination showed no significant aplasia and therefore, this presentation was explained by co-occurrence of GVHD and sepsis, of which this patient eventually died. The remaining patients showed no toxicities.

### Survival

**Figure 3.**
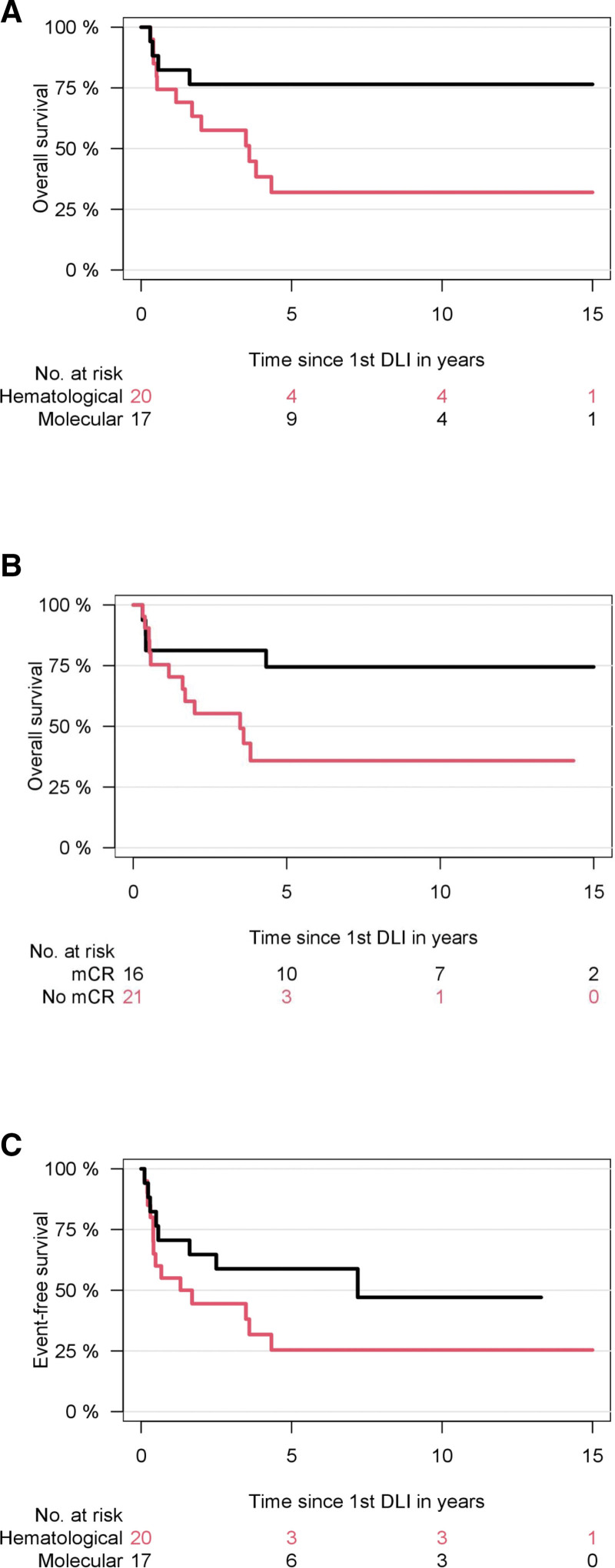
**Survival after DLI for relapsed myelofibrosis.** (A) Overall survival according molecular or hematological relapse. (B) Overall survival according molecular vs less than molecular remission after first DLI. (C) Event-free survival according type of initial relapse. All patients who relapsed after initial mCR to first DLI could be salvaged by another DLI exhibiting long-lasting mCR of a median of 7 years. DLI = donor lymphocyte infusion; mCR = molecular complete response.

Median follow-up from first DLI of the entire cohort was 6.1 years (95% CI, 1.7-10.5 years). OS according to the initial indication for DLI was 77% (95% CI, 57%-97%) for molecular relapse compared with 32% (95% CI, 10%-54%) for hematological relapse after HCT (*P* = 0.03). Of note, 6 patients received salvage second HCT after DLI failure, of whom all had hematological relapse after first HCT (Figure 3).

In terms of responders versus nonresponders, OS was 63% (95% CI, 43%-83%) versus 23% ([95% CI, 0%-50%]; *P* = 0.01). Outcome of responders versus nonresponders to first DLI was 75% (95% CI, 52%-97%) versus 36% ([95% CI, 13%-59%]; *P* = 0.04). Subsequent second allograft was a censored event. Outcome appeared to be impacted rather by initial relapse after HCT than by achievement of response (*P* = 0.05). Although responders appeared to have better outcomes, patients treated for molecular relapse appeared to have better outcome even for patients without initial response to first DLI in comparison with both responders and nonresponders treated for initial hematological relapse. Thus, OS for responders versus nonresponders to first DLI was 88% versus 68% for molecular relapse compared with 60% versus 12% for hematological relapse. Out of 12 total deaths in the hematological relapse cohort, 9 events occurred in patients who did not respond after first DLI, while 3 patients died, which showed mCR after first DLI for hematological relapse.

All 8 patients who relapsed after initial mCR to first DLI (out of 16 responders to first DLI, 50%) could be salvaged by another DLI exhibiting long-lasting mCR of a median of 7 years. Four patients had initial molecular relapse and 4 hematological relapse after HCT and are alive at last follow-up. Thus, OS was 100% versus 42% (95% CI, 16%-68%) for the 18 patients who did not show mCR after first DLI and who received subsequent salvage DLI.

Time to first DLI showed no significant association for OS (*P* = 0.14). No association between dose of first DLI and OS was observed (*P* = 0.79), while total number of DLI procedures appeared to be associated with longer OS in the entire cohort (*P* = 0.07). However, after adjustment for interaction with type of relapse after HCT (molecular versus hematological relapse), the impact of number of DLI was driven by type of relapse and not significant anymore (*P* = 0.33).

## DISCUSSION

Our study has 5 main findings. First, molecular monitoring enables to differentiate between molecular relapse after HCT, which is associated with significantly improved outcome in comparison with hematological relapse after HCT. Second, this approach can identify and treat relapsed patients early after HCT. Third, all patients who experienced subsequent relapse after having achieved mCR after first DLI could be salvaged with subsequent DLI, exhibiting long-term survival. Fourth, half of the patients achieve mCR without developing GvHD. Fifth, no patient that was initially treated for molecular relapse was in need of a second HCT as salvage strategy. Taken together, these findings demonstrate high efficacy with low GvHD incidence by escalating DLIs in relapsed myelofibrosis patients after allograft. Here, we suggest that applying molecular monitoring together with DLI should be standard of care for treatment of relapsed myelofibrosis after HCT.

The significant difference in outcomes between mCR and less than mCR suggests a potent graft-versus-myelofibrosis effect, which underscores the relevance for molecular monitoring and adoptive immunotherapy in myelofibrosis undergoing HCT. If patients do not respond to either escalating dose regimen or salvage DLI and relapse has occurred, then patient suitability for a second transplantation should be considered.^4,6^ However, this is often reserved for fitter, younger individuals with a good performance status who are felt to have sufficient reserve to undergo such an approach. In the present study, 6 patients received a second HCT and all belonged to the hematological relapse group. Two patients were alive at last follow-up, showing survival of 24 and 26 years since their first transplantation, respectively.

One important feature of our study is the uniform approach for conditioning and GvHD prophylaxis using ATLG, which is also most frequently implemented in transplantation platforms in Europe,^24^ while many US-based centers use non-ATLG-based GvHD prophylaxis.^25,26^ In a previous study comparing ATLG with non-ATLG approaches in the matched related donor setting, no significant difference was found in terms of disease-free survival, chronic GvHD, relapse-free survival, or relapse risk, while risk for acute GvHD was significantly reduced with ATLG.^27^ Other sufficient comparative analyses in other settings are still lacking. Whether our results presented here can be fully translated to other settings remains to be determined but should prompt investigations.

In terms of safety of DLI, low rates of acute and chronic GvHD were observed (22%, respectively) and were comparable with previous reports in other indications.^28,29^ One patient experienced grade IV acute GvHD after first DLI and died of sepsis and multiorgan failure. No clear correlation was found for DLI dose (either in total or per single dose) and GvHD, whereas only chronic GvHD appeared to be associated with overall number of DLIs (*P* = 0.01). However, 2 cases with severe aGVHD received a higher starting dose. On the contrary, response did not seem to correlate with DLI dose. Thus, our data suggest to avoid higher starting doses for first DLI and carefully escalate subsequent DLI in homogenous manner to safely apply this therapy without and achieve same efficacy.

A caveat of our study may be the focus on driver mutations for molecular monitoring. Importantly, patients with an *MPL* driver mutation genotype tend to show significantly lower rates of relapse and also better overall outcome.^9,17^ We included 4 triple-negative patients. Three patients achieved full chimerism and were alive at last follow-up. One triple-negative patient showed hematological relapse, never achieved full chimerism, and subsequently died. For these patients, novel methods based on the next-generation sequencing are currently under investigation to identify more patients at different risk for relapse or progression.^30^ We included sequencing data of 23 evaluable patients at the time of transplantation and found a slight absolute difference in distribution of high molecular risk in molecular relapse (27%) versus hematological relapse (58%). Another limitation of our study may be selection bias as molecular monitoring of patients was not standard practice for the whole study period. Information on molecular status was not available at the time of treatment for some patients who received first DLI for hematological relapse and samples were analyzed retrospectively in these cases. Thus, different time from relapse to DLI may have been confounded by availability of molecular analysis. However, the fact that patients with initial hematological relapse showed significantly worse responses, slightly higher rates of high molecular risk, and had to be salvaged by second HCT suggests a more aggressive disease, possibly affected by prolonged treatment initiation.

Last, we applied molecular monitoring in close sequences early after transplant, allowing for early detection but also requiring resources and technical capabilities that may not be generalizable. However, our finding that particularly patients with molecular relapse received DLI significantly earlier than patients with hematological relapse may underline the importance of early detection of relapsed patients for achieving excellent outcomes. Based on these results, it may be recommended to monitor patients early after transplant as closely as possible. In terms of sensitivity, we showed that donor chimerism correlated with molecular response after DLI and that the impact of chimerism analysis was mediated by type of relapse, while some patients showed mCR and excellent outcomes despite incomplete donor chimerism. This underscores the importance of molecular monitoring for best possible prognostic utility after DLI.

In conclusion, DLI for relapsed myelofibrosis after HCT showed excellent survival, particularly for patients with molecular relapse and who showed mCR at any time. Molecular CR can be achieved in half of the patients without development of debilitating GvHD. Furthermore, molecular monitoring enabled to treat early and to target mCR, even after several infusions, resulting in long-lasting response and survival. Our results underscore the need for molecular monitoring and targeting molecular response in relapsed myelofibrosis and provide evidence for DLI together with these approaches as a safe and effective strategy, which should be standard of care for these patients.

## AUTHOR CONTRIBUTIONS

NG and NK designed the study, collected and analyzed data, interpreted results, and wrote the first draft of the article. AB performed molecular monitoring. MG provided product collection. NG, CW, DJ, EK, FA, and NK infused the products and care for patients. CW, AB, MQ, DJ, CB, EK, MG, FA, and BF collected data, interpreted results, and wrote the article. All authors approved of the final version of the article.

## DATA AVAILABILITY

Data will be shared upon request via email to the corresponding author.

## DISCLOSURES

The authors have no conflicts of interest to disclose.
